# Management of Congenital Clinical Anophthalmos with Orbital Cyst: A Kinshasa Case Report

**DOI:** 10.1155/2018/5010915

**Published:** 2018-10-09

**Authors:** Thomas Stahnke, Andreas Erbersdobler, Steffi Knappe, Rudolf F. Guthoff, Ngoy J. Kilangalanga

**Affiliations:** ^1^Department of Ophthalmology, Rostock University Medical Center, Doberaner Straße 140, 18057 Rostock, Germany; ^2^Institute of Pathology, Rostock University Medical Center, Strempelstr. 14, 18055 Rostock, Germany; ^3^Eye Department, Hospital Saint Joseph, 322 Limete/Kinshasa, Democratic Republic of the Congo

## Abstract

An early developmental lack of the optic vesicle can result in congenital anophthalmia, defined as a complete absence of the eye, which can be distinguished from congenital microphthalmos, where ocular rudiments are present. Here, a rare pediatric case of congenital clinical anophthalmos with orbital cyst in the left orbit is reported. The patient was a 14-month-old girl with no other congenital defects who underwent surgical and prothetic management in St. Joseph's Hospital Kinshasa, Democratic Republic of the Congo (DRC). Surgery was carried out under general anesthesia. The cyst was punctured and its wall fully excised. Near the orbital apex pigmented elements representing iris, ciliary body, and choroidal or retinal remnants were found. The specimens were fixed in formalin for histological examination. Surgical cyst removal including socket deepening for an artificial eye was performed. Postoperative wound healing was uneventful and a satisfactory cosmetic outcome was achieved in all follow-up examinations. Histological examination revealed rudimentary ocular structures similar to degenerated lens tissue with a typical, PAS-positive capsule. Additionally, pigmented epithelial structures, which seem to be of ciliary body, iris, and choroidal or retinal-type epithelium origin, could be detected, prompting the final diagnosis, microphthalmia with dominant cyst formation.

## 1. Background

During development, ocular abnormalities of the eye include a wide range of malformations, depending on the embryonic age at onset of the disease [1]. One of these dysplasias is congenital anophthalmia, which is defined as a complete absence of the eye due to a developmental lack (primary anophthalmia) or an early differentiation arrest (secondary anophthalmia) of the optic vesicles during early phases of gestation [2]. Differentiation between primary and secondary anophthalmia as well as exclusion from severe microphthalmia (clinical anophthalmia), in which microscopic optic rudiments escaping clinical detection are present, is extremely difficult and can be determined by histological examinations only [2]. Congenital anophthalmia can affect one or both eyes during development leading to impaired visual capability or blindness, respectively. It is a rare disease with a prevalence rate of 0.3-0.6 per 10,000 births [3, 4]. Prevalence rates for a microphthalmos are higher and are ranging between 1.4 and 3.5 per 10,000 births [4]. A partial or complete failure in the involution of the primary optic vesicle during development results in the formation of a cyst, which persists and is found at birth to replace the globe of the eye [5]. Sometimes the cyst is small and the case presents clinically only as anophthalmos. In other cases the cyst can be quite large and may protrude the normal developed conjunctival sac and the eye lids [5]. The absence of one or two eyeballs, even if a cyst exists, is often accompanied with a deficient orbitofacial growth, leading to aesthetic deficits in congenital anophthalmia.

In industrial countries malformations like congenital anophthalmos and microphthalmos are usually treated as soon as possible in the first few weeks after birth to avoid serious aesthetic problems in the future provoked by an underdeveloped bony orbit as the result of the missing eyeball in utero. Different surgical implantation techniques like self-inflating orbita expanders [6] or socket expanders [7] have been developed to prevent deficient orbitofacial growth. Early orbita expansion is important to minimize facial deformity in cases of anophthalmia or severe microphthalmia [7]. The aesthetic aspect of anophthalmia or microphthalmia and its early therapy in industrial countries reduce the severity of medical and aesthetic problems.

In contrast, in developing countries and especially in rural areas where patients do not have access to standard medical care systems, the aesthetic aspect of congenital anophthalmos is of secondary importance. As a consequence, untreated cases of congenital anophthalmos are more common, which under given circumstances will not be present in industrialized nations.

Here we present the case of a clinical anophthalmos with an extensive orbital cyst in a 14-month-old African patient, who was finally treated during a foreign medical aid program.

## 2. Case Presentation

**Figure 1:**
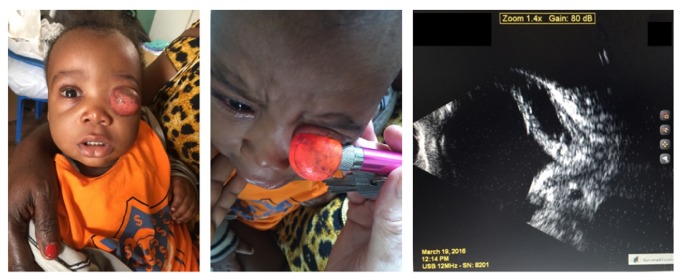
Patient with a clinical anophthalmos with orbital mass in the left orbit.** A:** protruded cyst.** B:** transillumination with a pen light revealed the cystic character of the structure.** C:** B-scan ultrasonography examination.

In the year 2000 the Rostock University Medical Center funded an ophthalmologic medical aid program in Kinshasa, Democratic Republic of the Congo. Over time, this funding developed to a close cooperation between Rostock and Kinshasa mainly focused on treatment of children with congenital cataract. Elective surgery outside this field was performed 1-2 times a year during visits of the Rostock team. At the last visit in Kinshasa in spring 2016, a Congolese mother of a 14-month-old female requested medical attendance for her daughter, who had a large orbital cyst with clinical anophthalmos in the left orbita since birth (Figure 1A). The mother reported that the pregnancy and birth were without complications. The child was born with a normal weight of 3.400 kg and the mother immediately noticed the orbital cyst in the newborn child. The mass did not increase in volume until the day of operation. The child was referred to St. Joseph's Hospital by an ophthalmologist for a specialized examination.

Visual inspection of the cyst disclosed a tissue mass, which protruded out of the left orbit. The tissue mass was nonpulsatile and irreducible. First examination by using a small pen light demonstrated transillumination, which indicated an expectation that it was filled with liquid (Figure 1B). The cyst was nontender and did not increase in size on coughing or crying. B-scan ultrasound examination demonstrated an orbital cyst with undefinable ocular tissue structures or underdeveloped optic rudiments (Figure 1C). There were no other congenital defects in the patient; the mother provided the information that the gestation was uneventful.

## 3. Surgery and Follow-Up

**Figure 2:**
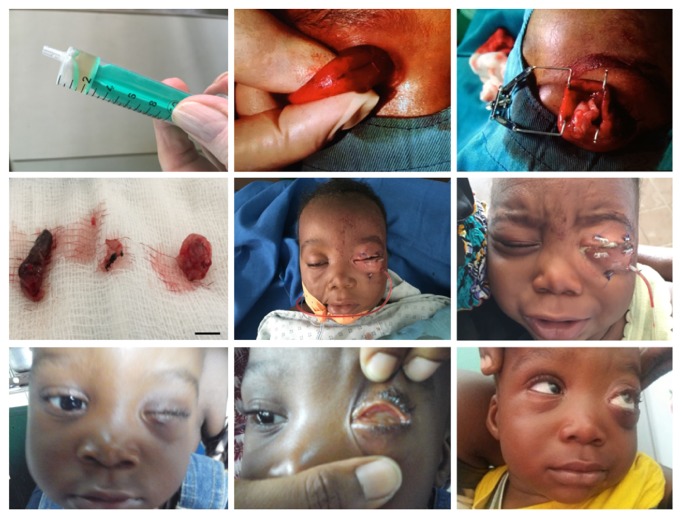
Intrasurgical photodocumentation and follow-up.** A:** drained cystic liquid;** B:** decompressed cyst;** C:** opened cyst;** D:** excised tissues; bar: 5 mm;** E, F:** transcutaneous fornix deepening sutures fixed over bolsters;** G, H:** 6 months' follow-up;** I: **8 months' follow-up.

The procedure was carried out under general anesthesia and well tolerated by the patient. In a first step the cyst was punctured and 1.5 ml of liquid was drained (Figure 2A). After drainage, the decompressed cyst appeared flabby shape (Figure 2B). In a second step the cyst was widely opened and the rims were fixed by a lid speculum (Figure 2C).

Undefined tissue structures were excised (Figure 2D). The cyst wall was split to remove the internal lining. After wound closure (4-0 prolene) transcutaneous fornix deepening sutures were placed and fixed over bolsters. Finally, a PMMA-conformer was inserted and the lid margin was temporarily sutured together with prolene (4-0). The removed specimen material (Figure 2D) was fixed in formalin.

The patient recovered well from the surgical intervention and sutures were removed 14 days after surgery. Six months after surgery the child was doing well and the examination of the orbit showed no complications (Figures 2G and 2H). Two months later, an acrylic prefabricated artificial eye was placed in the socket (Figure 2I) but the mother finally preferred to keep the child without any prosthesis. Up to the present, the mother still refuses to maintain the prosthesis in the socket.

## 4. Histology

The surgical specimens were submitted in formalin and transferred to the Institute of Pathology, Rostock University Medical Center.

Macroscopic inspection disclosed several membranous tissue specimens with diameters up to 1.5 cm. One of these specimens contained a small, partly calcified nodule, measuring 2 mm.

**Figure 3:**
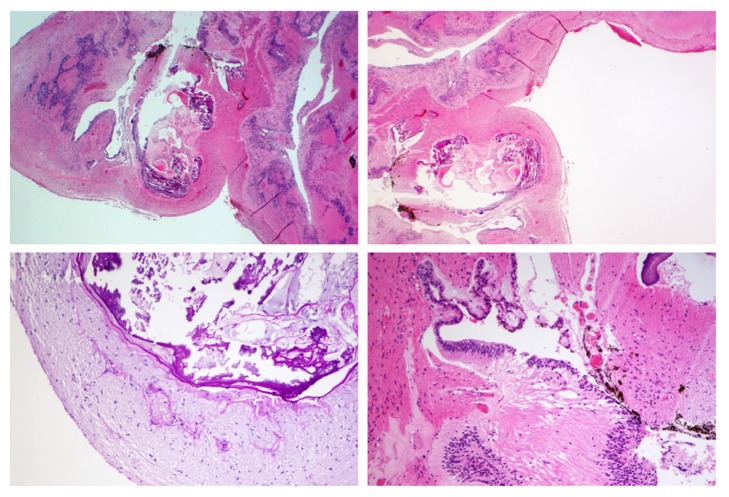
Histological examination of intracystal tissue. Magnification** A**,** B**: 20x;** C**,** D**: 100x.** A**,** B** and** D**: haematoxylin and eosin (H&E) staining;** C**: PAS staining.

Histologic sections showed the wall of a pseudocyst, consisting of fibrous tissue (Figures 3A and 3B). The calcified nodule was reminiscent of a degenerated lens with a typical, PAS-positive capsule (Figure 3C). Adjacent to the nodule, pigmented epithelial structures could be found, which is reminiscent of the ciliary body, and finally merged with retinal-type epithelium (Figure 3D).

Based on the histological findings a pathological diagnosis of a microphthalmia with dominant cyst formation was made.

## 5. Discussion

Here, we present a case of a 14-month-old girl with microphthalmos with a prolapsing, lid spreading cyst from the left orbit. Transillumination and B-scan ultrasonography strongly supported this diagnosis. Final confirmation was made after excision by histological examination and based on existing rudimental ocular structures. As far as the family history is determined this child is the only one in this family with such an ophthalmological defect. None of the other family members have had a similar eye problem before. No other nonophthalmic deficits or malfunctions were obvious and the physical and mental development of this child was within normal limits for her age. After cyst removal through surgical intervention and dispensing of a prosthesis, a satisfactory cosmetic result was achieved.

The failure of fetal fissure closure during ocular development in embryogenesis can result in orbital and ocular malformations including congenital cystic eye (“anophthalmia with cyst”) and microphthalmos with cyst (“colobomatous cyst”) [8–11]. Both are very rare abnormalities, although the last mentioned is much more common than the congenital cystic eye. The term congenital cystic eye was first used by the ophthalmologist Ida Mann [12]. The etiology of the cystic eye remains unclear; a genetic disposition is assumed [11, 13–16]. A frequent presence of inflammatory cells in the cysts suggests also a possible inflammatory etiology [17].

For reasons that are not entirely clear the congenital cystic eye develops as a result of partial or complete arrest in the invagination of the primary optic vesicle between the 2 mm and 7 mm stages of fetal development. In contrast, microphthalmos with cyst develops as a result of disturbances during later stages (7 mm to 14 mm), when the optic vesicle has already merged with the surface ectoderm and intraocular elements such as the lens or other ocular structures such as the cornea can be present [9, 10, 18, 19].

A distinguishing feature between these two malformations is the existence of a small eye or rudimentary ocular tissues in the microphthalmos with cyst that sometimes are difficult to visualize. Clinically, it may also appear as an eye with invisible cyst, as an obvious cyst with a small malformed eye or as a huge cyst, which can displace the eye and fills out the whole orbit [17]. In this case the cyst usually pushes out the lower lid because the cyst is attached to the inferior portion of the globe. In this form it can be identified easily as a variably sized, soft, and bluish orbital mass, which is normally filled with serous fluid. In the majority of cases with a congenital cystic eye the cyst normally protrudes under the central or upper eyelid or it prolapses between the eyelids.

Cysts in both malformations are benign structures and can occur unilaterally as well as bilaterally. It is also possible that both forms of cystic eyes can occur in the same patient, but in the majority of children with a congenital cystic eye the fellow eye is normal [9, 10, 13, 16]. Interestingly, in most published cases of congenital cystic eyes, the left eye has been the one affected [9, 11, 14–16], similar to the case presented here.

In some cases, most often in bilateral presentations, additional systemic/nonocular defects such as clef lip or basal encephalocele, agenesis of the corpus callosum, midbrain deformity, microcephalus, and saddle nose have been observed. To establish the correct diagnosis of these malfunctions, recognition of the clinical features, imaging techniques, and histopathologic findings are necessary. Histological examinations can differentiate between congenital anophthalmos and microphthalmos and are important for gaining information regarding insights of the pathogenesis (malformation vs deformation), and to help determine the prevalence of each entity.

Imaging diagnostic tools like ultrasonography, CT scan, or MRI not only are useful in the diagnosis of this rare entity, but also can be helpful in the identification of other associated brain abnormalities. In general, imaging should be performed preoperatively, because meningocele and optic nerve sheath cysts should be ruled out [9, 14]. Unfortunately, such preventative measures are available, predominantly in industrial countries. In developing countries, however, such examinations are limited due to a lack of the necessary medical equipment and the low income for a large section of the population.

There is no standardized protocol for managing a congenital cystic eye [20]. A volume reduction by cyst puncturing or surgical interventions like complete cyst removal with insertion of an orbital expander, followed by prosthetic fitting to receive an optimal cosmetic result, are indicated [6, 21, 22]. Such complicated interventions with complex follow-up procedures are, unfortunately, available only in industrial countries. In developing countries, however, surgical intervention as a treatment for a congenital clinical anophthalmos with orbital cyst is still an important effort if only to increase a patient's acceptance in society, even if preoperative diagnostic measures and the supply of artificial eyes are limited.

## 6. Conclusion

Surgical intervention of congenital clinical anophthalmos with orbital cyst is desirable to increase patient's acceptance in society, with or without adaptation of an artificial eye. By performing histological examinations it is possible to differentiate between congenital anophthalmos and microphthalmos. This is important for gaining information regarding insights of the pathogenesis and to learn about prevalence rates of each entity.

## Data Availability

All data gathered during this case report, which is only in form of photographs taken during the surgery, and follow-up visits, as well as histological sections, are presented within this manuscript. The original image files are available from the corresponding author upon reasonable request. Further data, aside from personal information regarding the patient, was not gathered. This information cannot be disclosed to guarantee patient anonymity.
